# Designing mHealth Apps for Substance Use Recovery Through Real-World Co-Design and Deployment: Mixed Methods Study

**DOI:** 10.2196/83984

**Published:** 2026-02-11

**Authors:** Teale Masrani, David C Hodgins, Hyoun S Kim, Katherine Rittenbach, Erika Johnson, Geoffrey Messier

**Affiliations:** 1 Department of Electrical and Software Engineering Schulich School of Engineering University of Calgary Calgary, AB Canada; 2 Department of Psychology Faculty of Arts University of Calgary Calgary, AB Canada; 3 Department of Psychiatry Cumming School of Medicine University of Calgary Calgary, AB Canada; 4 Department of Psychology Faculty of Arts Toronto Metropolitan University Toronto, ON Canada; 5 Department of Community Health Sciences Cumming School of Medicine University of Calgary Calgary, AB Canada; 6 Department of Clinical Neurosciences Cumming School of Medicine University of Calgary Calgary, AB Canada

**Keywords:** co-design, digital health, mHealth, smartphone application, substance use disorder

## Abstract

**Background:**

Mobile health (mHealth) apps have shown promise to support recovery from substance use disorders. However, evidence on engagement and efficacy is still inconclusive.

**Objective:**

This study aims to identify design considerations for optimizing engagement in mHealth apps for those recovering from problematic substance use, by analyzing real-world experiences with co-designed app features.

**Methods:**

We co-designed, deployed, and evaluated an mHealth app. Initial co-design interviews with 14 individuals in recovery led to 3 new features integrated into an existing mHealth app. The app was deployed for a 6-week trial with 53 participants using it during their daily routines without researcher supervision. Usage patterns were analyzed throughout the trial period, and follow-up interviews with 12 app users foregrounded subjective usage experiences and considerations for future design.

**Results:**

We developed 3 new features following co-design interviews: a goal-setting feature, a craving tracker, and a meetings log. Usage metrics revealed mixed engagement, with 45.3% (24/53) of users actively engaging with the app throughout the trial. These active users opened the app 27.1 unique times on average, with a retention rate after 30 days among active users of 45.8% (11/24), exceeding the typical mobile app retention benchmark of 7% after 30 days. Interviews revealed that participants preferred app functionality to extend beyond substance use domains to support other dimensions of their lives not directly pertaining to substance use, such as general goals and daily routines. Participants further suggested that recovery apps should act as private digital journals while also providing a sense of community and connection to broader recovery ecosystems. Additionally, mHealth designs that allow users to configure their own personalized recovery pathways in the app can benefit some users who appreciate increased autonomy, while others may become overwhelmed by a lack of prescriptive guidance.

**Conclusions:**

It is valuable to incorporate iterative co-design methodologies into digital health and recovery app research to help optimize engagement. Furthermore, recovery apps can benefit from flexible designs with customizable degrees of user autonomy. Future designers can better cater to individual user preferences by personalizing mHealth designs so that they strike a balance between system control and user control over digital recovery pathways.

## Introduction

Mobile health (mHealth) apps have become popular tools to manage personal health, offering inexpensive and accessible health guidance, self-monitoring of progress, and interactive communication with clinicians, among other capabilities [1]. A major aspect of some individuals’ personal health is use of substances, and recovery from disordered substance use. “Recovery” is defined by the individual and can have a variety of meanings, from reducing problematic use to practicing full abstinence [2,3]. Specialized mobile health apps have become popular tools to facilitate all forms of recovery, with many commercially available in iOS and Android app stores [4-6].

Prior research finds some potential for mHealth apps to facilitate recovery [7-10]. However, the effectiveness and engagement of these interventions remain unclear [8,9,11-13]. Effects are often minimal, or only notable when the app is used in conjunction with other non-app-based treatments [14-16]. Furthermore, many studies in this field use a randomized controlled trial methodology. These studies often compare any usage of the app to treatment-as-usual, and tend to overlook the details of how users engaged with particular app features and their motivations behind specific engagement patterns [8,12,17-19]. Therefore, some researchers argue that studies “should not assess the success of addiction recovery apps based only on objective measures of changes in substance use” [12], and should instead aim to understand users’ subjective experiences when using such tools as a way to evaluate quality.

This body of research may additionally benefit from a greater focus on the designer’s point-of-view, unpacking the details of how digital recovery tools can be designed to best support their users [17,18], rather than only studying user outcomes after the app has already been built and deployed. Careful user-experience design and research play key roles in the success of mHealth apps [12,19], as they can involve human-centered techniques such as value-sensitive design [20,21] and participatory or collaborative design (co-design) [22-24]. In the field of mobile recovery apps, however, such human-centered design techniques are scarce [13]. This study incorporates the co-design and deployment of an mHealth recovery app to investigate the real-world experiences of people dealing with problematic substance use. In doing so, we shed light on practical design considerations to optimize app engagement among this population.

There is a small but growing body of research that shares the aim of centering end users’ perspectives to design engaging mHealth recovery apps [25-28]. One prior study is particularly relevant: Jones et al [29] investigated how individuals engaged with an early version of a self-tracking app, identifying considerations for future designs. Similar to our work, Jones et al deployed a working prototype of the tool to stimulate discussion with participants and gather mixed methods usage data. Notably, Jones et al [29] focused only on features that support day-to-day activity tracking, discussing themes pertaining to the types of activities participants tend to track and how they preferred to track them. Herein, we used similar methodologies in pursuit of similar aims, but importantly expanded beyond activity tracking to also investigate the design of goal-setting features and several other mHealth feature types.

Specifically, our research objective was to gain a feature-level understanding of how to create an mHealth app that engages people on their recovery journey. We achieved this objective by co-designing new app features and examining how individuals in recovery perceived and interacted with them during their day-to-day lives over a 6-week period. We took a mixed methods approach to evaluate both quantitative usage metrics and qualitative accounts of users’ experiences with the new features.

## Methods

### Overview

We adopted a research through design (RtD) approach [30-32]. RtD involves conducting research that “employs the methods, practices, and processes of design practice, with the intention of generating new knowledge” [31]. RtD encompasses the hands-on practice of designing a new tool, evaluating its functionality, and reflecting on its design to identify implications for future technologies. In accordance with this hands-on approach, we collaborated with the developers of an existing mHealth app, Zamplo [33], to complete three phases of this work: (1) initial co-design interviews; (2) design, deployment, and a 6-week app trial; and (3) user-experience interviews.

### The Zamplo mHealth App

Zamplo’s existing functionality does not cater specifically to substance use recovery, as it is used as a general mHealth tool. Users of mHealth technologies tend to face difficulties using a suite of different apps with several overlapping and fragmented features, often preferring a single, flexible unified platform instead [34]. Zamplo aims to overcome these issues with an array of flexible digital health capabilities within one platform to support multiple aspects of personal health. Existing Zamplo capabilities include customizable reminders, daily personal tracking, and visualizations of medications and activities, as well as tracking of mental and physical health symptoms, and management of health documents, to-do lists, notes, and important contacts.

For this study, we configured these features to not assume the preferences of users, such as what specific health metrics they may want to track, what types of goals they will set, or how frequently they prefer to receive reminders. Instead, we configured Zamplo as an open-ended platform, allowing users to self-track, set goals, create reminders, and access several other mHealth features, in whichever ways they choose, without enforcing any planned usage patterns. This open-ended approach allowed for naturalistic observations of how individuals organically use mHealth features when not pushed to engage in a particular way.

We co-designed new features to add to the Zamplo platform and used Zamplo as a technology probe. Hutchinson et al [35] define technology probes as technological artifacts that guide researchers in understanding their users in a real world setting and inspire participants to think creatively about new potential designs [35]. Hence, Zamplo was used as a concrete artifact to ground participants’ interviews, stimulate discussions of users’ experiences, and crystalize users’ needs. It was a particularly well-suited technology probe as it was created and approved for the secure storage of health data, allowing extensive possible usages in the trial than available in most mHealth apps. This research consisted of three phases, as outlined below.

### Phase 1: Initial Co-Design Interviews

#### Participants

This research began with an initial round of interviews to capture design ideas from individuals with experience recovering from substance use disorders. Participants were recruited from 5 local addiction treatment centers. Of the 23 individuals who signed up to participate, 14 attended an interview. Informed consent was obtained via an electronic consent form and CAD $20 (USD $14.76) electronic gift cards were offered to all interviewees.

#### Procedure

The research team collaboratively developed a semistructured interview protocol, and 45-60 minute interviews were conducted virtually by one interviewer. Participants were first asked about their recovery history and experiences using technology. This contextualized the primary component of each interview, which was a demonstration of the existing Zamplo platform, followed by inquiry into participants’ impressions and ideas for future designs.

Interviews revealed participants’ stated needs regarding mHealth technologies and their perceived role in recovery. While the interview protocol provided a framework for discussion topics, the direction of each interview was guided by participants [36]. As such, participants were encouraged to speak openly and creatively about their ideas for Zamplo and were prompted to provide both positive and negative feedback. To mitigate social desirability bias, the interviewer clearly stated that the app was not their own and that all critiques are welcome.

#### Data Analysis

Interviews were recorded using Zoom (Zoom Video Communications, Inc) and transcribed using Rev AI (Rev.com, Inc) transcription software. The interviewer then manually reviewed and corrected each transcript, before manually analyzing transcripts using the NVivo (Lumivero) platform. A generative open-coding approach was taken for each transcript, iteratively generating, refining, and categorizing original codes throughout analysis [37]. The goal of coding was to identify actionable design implications for new Zamplo features. Several design themes based on the interviews were identified and collaboratively refined via discussions with the research team. These results were relayed back to Zamplo developers to implement, launching the next phase of the research.

### Phase 2: Design, Deployment, and 6-Week App Trial

#### Overview

Interview themes from Phase 1 laid the foundation for creative co-design discussions with the research team and the lead Zamplo developer. Discussions resulted in several low-fidelity wireframe-style prototypes [38] of potential new features, based directly on input received from participants. A new trial version of Zamplo was then deployed on iOS and Android app stores, and recruitment opened. There were two goals for this trial: first, to gather quantitative usage metrics to evaluate usage patterns and engagement; second, to provide participants with a concrete mHealth app experience to stimulate productive reflections during Phase 3 interviews. The following sections detail the methods of this trial.

#### Participants

Participants were recruited through posters in several treatment centers and online advertisements. Screening excluded those who were not in Canada or were not currently attempting to stop or reduce their substance use. Informed consent was obtained via an electronic consent form, after which they were provided a direct link to download Zamplo and begin their 6-week trial.

#### Procedure

This was a single-arm trial in which all participants were given the same mHealth intervention. Recruitment occurred from November 29, 2024, to January 17, 2025. After creating a Zamplo account, participants navigated through a text-based tutorial of the features available to them. The tutorial encouraged participants to explore all features and use the app in ways that felt most natural to them. They were further informed that they were not required to use the app to receive their monetary incentive, and all data they entered into the app will be kept entirely private. Participants were provided with an email address for further technical help, if needed. Overall, participants were left to independently engage with the app during their regular lives.

When participants reached the end of their 6-week trial, the app directed them to a final survey to provide information about their demographics and substance use history, rate the helpfulness of the app, and provide consent to be contacted for a follow up interview. They were then offered a CAD $20 (USD $14.76) electronic gift card for their participation. Those who did not submit a final survey after their trial period were considered to have dropped out of the study and were not included in the final analysis.

#### Data Analysis

Data were collected on the number of times participants opened the app, the duration of each app usage session, and the frequency with which participants used each individual feature. These data informed descriptive statistical analyses of engagement. Furthermore, participants were divided into categories based on demographics (gender, age, and ethnicity), recovery goals (abstinence or harm reduction), most problematic substance (alcohol, cannabis, or other illicit substances), and perceptions of Zamplo’s helpfulness. Engagement was compared across groups.

### Phase 3: User Experience Interviews

#### Overview

To gather further insight, we conducted follow-up interviews with a subset of Phase 2 app users, inquiring about their experiences with the app and suggestions for future designs. These qualitative interviews added nuance to quantitative app usage metrics. While Phase 2 usage metrics provided valuable information on how frequently each feature was used, interviews shed light on *why* participants chose to use or not use particular app features, and other subjective experiences with the platform.

#### Participants

After participants completed their 6-week app trial, 42 consented to be recontacted for a follow-up interview. Researchers contacted 22 of these participants to schedule interviews. Others were not contacted due to suspected fraudulent participation, zero app usage during Phase 2, or not engaging meaningfully with the final survey, such as by skipping all questions. Of these 22 participants, 12 attended an interview. Informed consent was obtained via an electronic consent form, and CAD $20 (USD $14.76) electronic gift card incentives were offered to all interviewees.

#### Procedure

Interviews were 45-60 minutes in duration and conducted virtually by the same interviewer as in Phase 1. Interviews consisted of 3 main types of questions: the participant’s history using apps in general during their recovery, their experiences with Zamplo during the app trial, and questions prompting the participant to brainstorm about “ideal” mHealth app functionalities to support their recovery. Both positive and negative feedback were encouraged.

#### Data Analysis

Interviews were recorded using Zoom, and transcribed using Rev AI transcription software. Each transcript was manually reviewed and corrected by the interviewer.

Transcripts were manually analyzed using the NVivo (Lumivero) platform, and coding was completed in 3 stages. The purpose of the first stage was to generate a codebook [39]. To do so, the interviewer conducted a preliminary round of coding. Coding was inductive [36], and after becoming familiar with transcript data and noting initial impressions, the coder generated original codes using an open-coding process [37]. Several low-level preliminary codes were created and clustered into a hierarchical structure. High-level categories were then refined to create a codebook based on collaborative discussions among the research team. A copy of the final codebook is available in Multimedia Appendix 1.

The initial coder and a second coder then used the codebook to code the full set of transcripts, making up the second stage of analysis. Both coders coded the same first transcript, then met to discuss coding disagreements and refined the codebook based on consensus. They repeated this process with a second transcript. After all remaining discrepancies in their interpretation of the codebook were resolved, each coder was randomly assigned 5 of the remaining transcripts to independently code using the codebook. Coding focused on capturing meaning as communicated by the participants (semantic coding), rather than attempting to identify implicit meaning behind participants’ words (latent coding) [36,37].

Once all transcripts were coded using the codebook, the interviewer conducted a final thematic analysis of the coded transcripts, comprising the third stage of analysis. This final, high-level analysis generated meaningful themes with accompanying illustrative quotes to characterize how participants experienced Zamplo, and their values regarding mHealth apps for recovery.

### Ethical Considerations

All phases of this work were approved by the University of Calgary Conjoint Faculties Research Ethics Board (REB24-0015). Informed consent was obtained via electronic consent forms for each phase. CAD $20 (USD $14.76) electronic gift cards were offered to all interviewees; participants were not required to meet any app engagement thresholds to receive the incentive. Participants were informed that Zamplo usage data were completely anonymous and confidential, and that survey data would be kept confidential with identifying information stored separately from deidentified survey data using anonymous participant identifiers.

## Results

### Phase 1: Co-Design Results

#### Overview

A total of 14 individuals participated in the initial co-design interviews. The mean interview duration was 58.9 minutes, with the longest interview lasting 107 minutes and the shortest lasting 47 minutes (13.7 hours in total). Themes from the initial interviews described several design implications for new Zamplo features. After iterative co-design discussions, 3 features were deemed feasible and worthwhile: a goal-setting feature, a craving tracker, and a meetings log. Features that were not included due to technical or resource constraints were a sobriety tracker and a mood tracker. Sobriety can instead be tracked within the goal-setting feature, and aspects of mental health can be tracked with existing Zamplo features upon user customization. The following sections detail the design and rationale of each new feature.

#### New Feature 1: Goal-Setting

**Figure 1:**
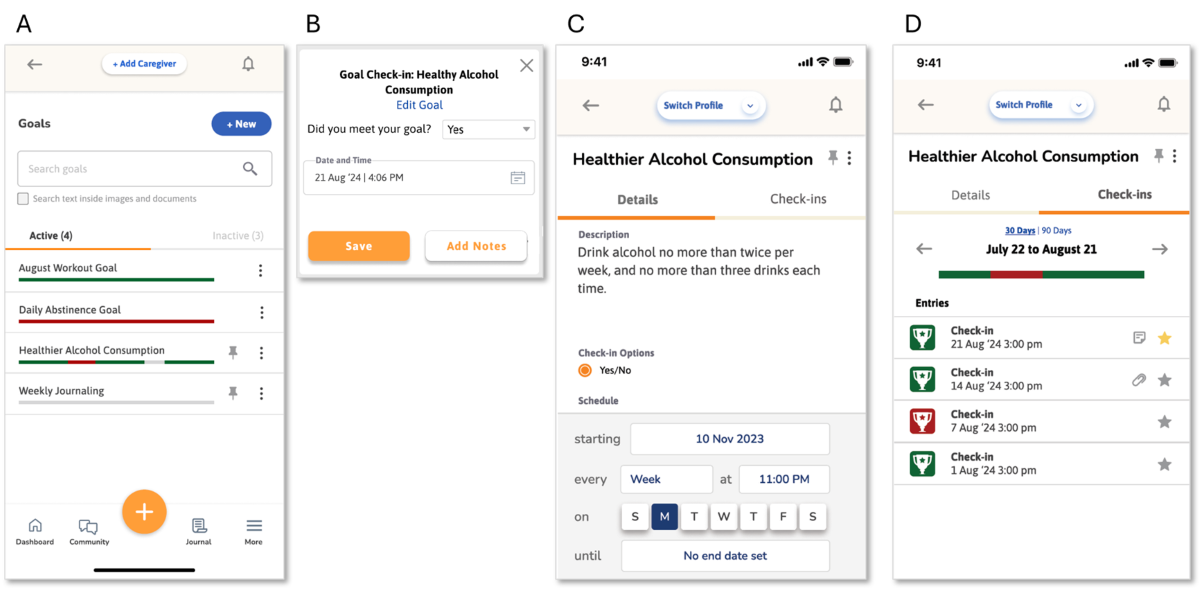
Goals feature mock-ups. (A) All active goals displayed with current check-in progress. Red bars indicate negative check-ins; green bars indicate positive check-ins. (B) An example of a scheduled goal check-in notification for one goal, asking if the user met their goal, with a selected option (“Yes”) and a date and time. (C) Viewing or editing one particular goal, including the title, description, and customized check-in schedule. (D) Detailed history of goal check-ins for one particular goal.

During co-design interviews, participants emphasized that seeing ongoing progress of their recovery is crucial for their motivation. A visual anchor can keep them focused on and accountable to their recovery goals. However, they also described feeling frustrated and demoralized by existing sobriety tracker apps that reset and erase all previous data upon a relapse. This gave rise to the design idea of a nonconsecutive goal tracker that does not add more feelings of shame to relapses and allows imperfect adherence to goals. In contrast to sobriety trackers that only track consecutive sober days, this feature acknowledges missed days without resetting the tracker. Users can create any new goal to track, modify a goal at any time, create a flexible goal end date, and customize time intervals for regular “check-in” notifications (daily, weekly, or otherwise). At the user-specified check-in intervals for each goal, the feature generates a goal check-in notification, where users select “Yes,” “No,” or “Somewhat” in response to the question “Did you meet your goal?” A visual progress bar is gradually created over a 30- or 90-day timeframe for each goal, reflecting the history of the user’s goal progress according to their check-ins. The progress bar displays both successful and unsuccessful days. Figure 1 shows final mock-ups of this feature. Importantly, this feature can be used to set and track goals outside of substance-use as well.

#### New Feature 2: Craving Tracker

Interview analysis also revealed a desire to track cravings, along with triggers and solutions for each craving and additional health data. Participants explained that they would benefit from tracking cravings alongside several other metrics to understand potential reasons for the craving. For example, P008 described what they would like to see in a future iteration of Zamplo:

It'd be kind of cool to track when you're getting cravings, and then you can determine why you got that craving. Maybe because your physical activity was down, maybe because you stopped going to meetings, or maybe you're doing too many meetings, or too much physical activity or too much work. So, it'd be cool to track your balance that way so you can figure out where you go wrong.

P008

**Figure 2:**
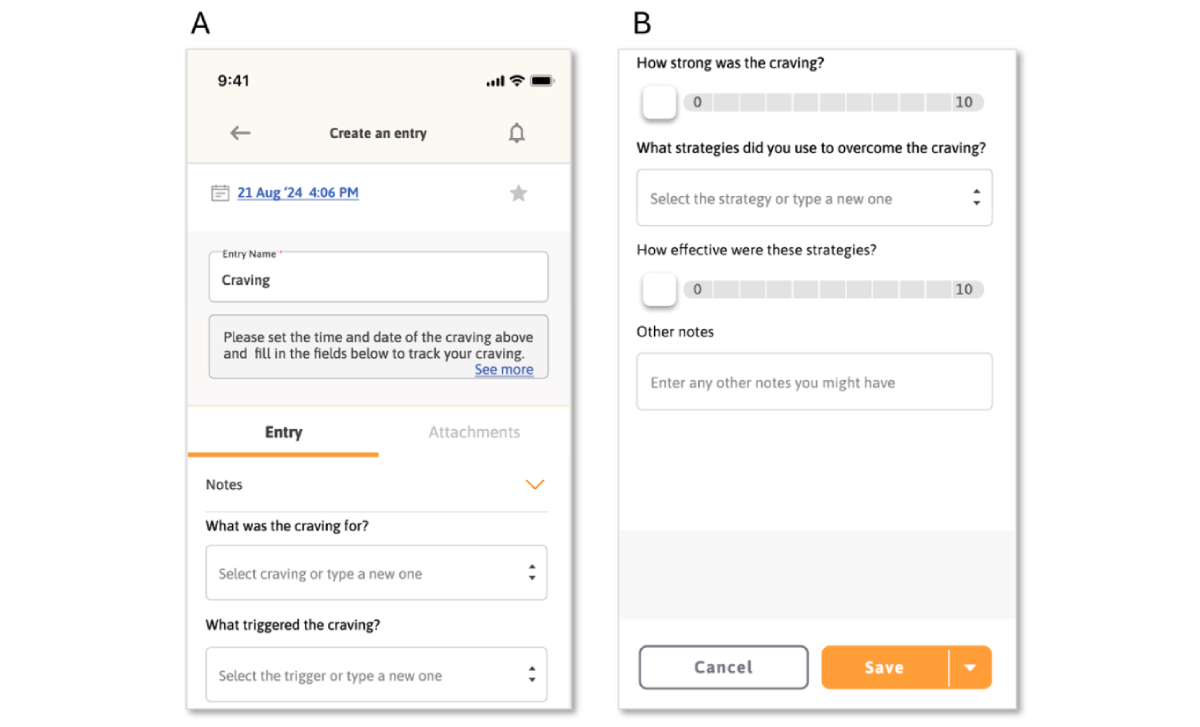
Craving tracker mock-ups. (A) The top of a new craving entry page. (B) The bottom of the entry page (scrolled down).

These types of participant sentiments led to the design of the craving tracker feature (Figure 2), which allows users to log details of each craving. The craving tracker is shown prominently on the main app dashboard for users to tap any time they experience a craving.

#### New Feature 3: Meetings Log

The final feature that arose from initial interviews was a meeting log. Participants described the importance of keeping track of meetings, appointments, and other treatment sessions. Participants not only needed reminders for meetings, which they can create using Zamplo’s existing activity tracker, but they also wanted to log the details of each meeting to revisit in the future. P004 describes how tracking Alcoholics Anonymous meetings can boost their motivation:

If you documented your meetings list, you would see, ‘wow, I went to a hundred meetings in the last 90 days,’ or something like that. It shows how much time and effort you put in.

P004

**Figure 3:**
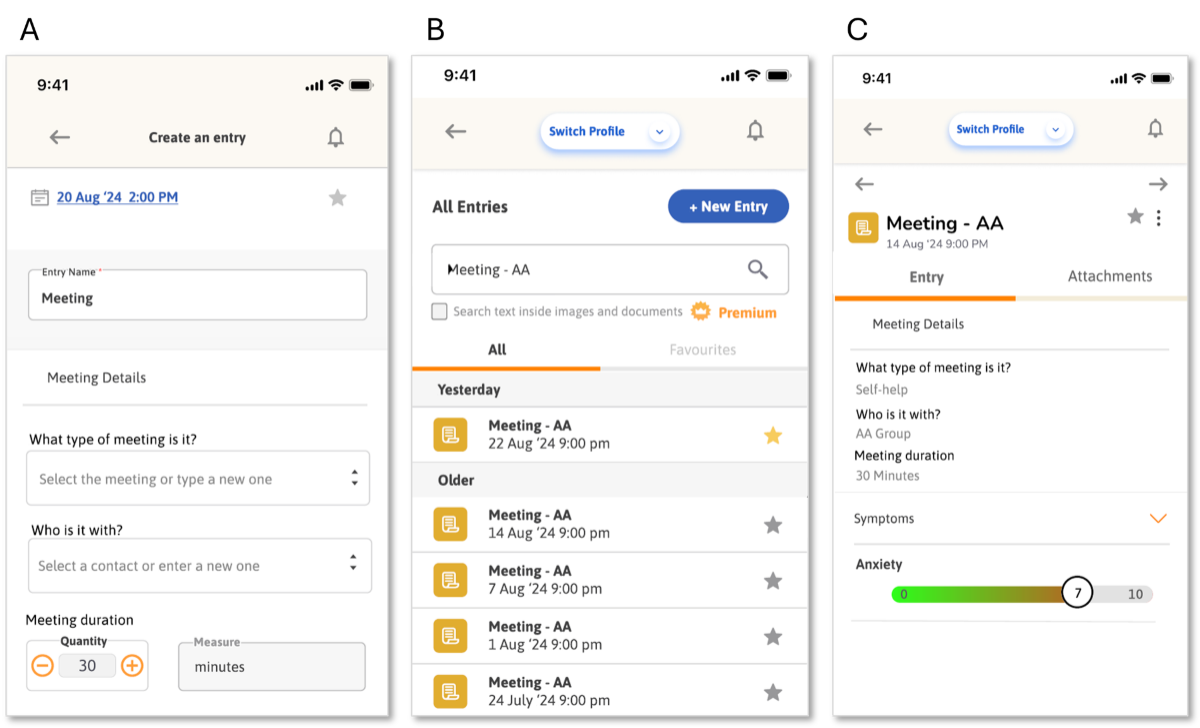
Meetings log mock-ups. (A) A portion of the new meeting entry page. Unshown fields include, for example, notes, mental and physical symptoms, and to-dos. (B) History of all past meeting entries. (C) Viewing the details of a previous meeting log.

Figure 3 depicts final mock-ups of the meetings log feature.

### Phase 2: App Usage Patterns During the 6-Week Trial

**Figure 4:**
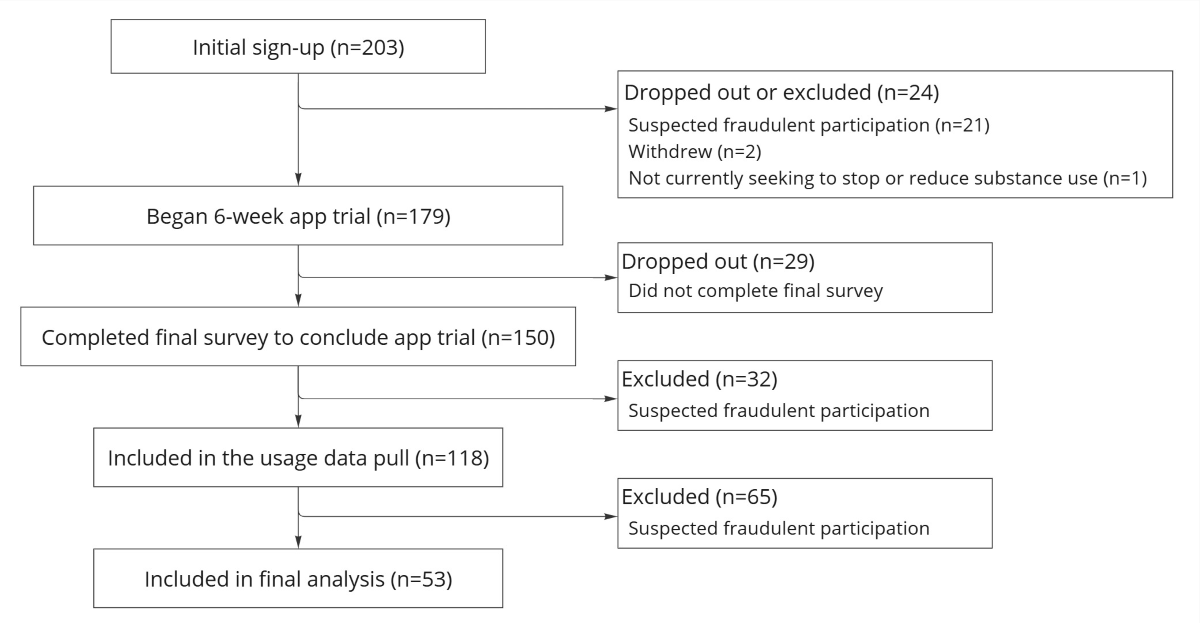
CONSORT (Consolidated Standards of Reporting Trials) diagram for Phase 2.

Figure 4 contains a CONSORT (Consolidated Standards of Reporting Trials) flow diagram of participation throughout this single-arm trial. Due to widespread online recruitment channels, fraudulent participation posed issues for this second research phase. Therefore, we established several authenticity screenings that effectively screened out fraudulent participation.

Of the 203 accounts initially created, 21 were suspected fraudulent due to circumventing the mandatory screening questionnaire meant to be completed before account creation. A further 32 participants were suspected fraudulent after completing their final survey after the trial, due to mismatched demographic responses when compared to initial screening, such as their age decreasing. The Zamplo usage data pull included 118 accounts. However, several accounts shared the same device address, indicating a possibility that each account did not represent a unique person. Accounts were designated *authentic* if they did not share an address with other accounts. As shown in Figure 4, a total of 65 potentially fraudulent accounts were detected using this approach. The final sample size of authentic participants was 53.

App users were divided into several subcategories based on demographics, recovery approach, most problematic substance, and ratings of the app’s helpfulness. These groups provide important context for design, as they demonstrate which sectors of the community found the app most useful. The final numbers of authentic users in each group are shown in Table 1.

**Table 1:** App user groups, with numbers of authentic and active users.

Group			Authentic users, n/N (%)		Active users^a^, n/N (%)
All users			53/53 (100)		24/53 (45.3)
**Gender**					
	Women	29/53 (54.7)		15/29 (51.7)	
	Men	23/53 (43.4)		8/23 (34.8)	
**Recovery goal**					
	Abstinence recovery goals	36/53 (67.9)		14/36 (38.9)	
	Harm reduction recovery goals	17/53 (32.1)		10/17 (58.8)	
**Perceived app helpfulness**					
	Somewhat to very helpful	35/53 (66)		13/35 (37.1)	
	Somewhat to very unhelpful	12/53 (22.6)		9/12 (75)	
**Most problematic substance**					
	Alcohol is most problematic substance	18/53 (34)		9/18 (50)	
	Cannabis is most problematic substance	14/53 (26.4)		5/14 (35.7)	
	Other most problematic substance	21/53 (39.6)		10/21 (47.6)	
**Age group (years)**					
	18-29	14/53 (26.4)		5/14 (35.7)	
	30-39	21/53 (39.6)		14/21 (66.7)	
	40-59	11/53 (20.8)		4/11 (36.4)	
**Race and ethnicity**					
	White	32/53 (60.4)		14/32 (43.8)	
	BIPOC^b^	18/53 (34.0)		7/18 (38.9)	

^a^A user was considered “active” if they opened the app on 2 or more separate days, excluding the first and last days of the study period.

^b^Black, Indigenous, and People of Color.

The designation of an *active* user was created to ensure that usage data were analyzed from those who meaningfully interacted with the app. An active user was defined as someone who used the app during at least 2 separate 24-hour intervals. Usage in the first and last 24-hour periods of a person’s trial did not count toward the active user designation, since those intervals included activity required for minimal study compliance. Specifically, the first 24 hours involved downloading the app to begin the trial, and the last involved following a link to conclude the trial. The number of active users in each demographic group are shown in Table 1.

While many of the group sizes in Table 1 are small, they allow for preliminary comparisons between groups. A greater proportion of women actively used the app (15/29, 51.7%) compared to men (8/23, 34.8%). Overall, the greatest number of active users were women (n=15), followed by those aged 30-39 years (n=14), those who identified as White (n=14), and those who aimed to fully abstain from substance use (n=14). However, a higher proportion of users taking a harm reduction approach to their recovery actively engaged with the app (10/17, 58.8%) compared to those taking an abstinence-based approach (14/36, 38.9%).

Table 2 depicts how often the active users in each participant group opened the app over the 42-day trial period. A “session” was logged by Zamplo each time a user opens the app for any reason, either to enter data or simply to view information or explore the interface. Table 2 includes the mean (SD), median (IQR), and top 25th percentile number of sessions for each group.

**Table 2:** Number of sessions among active users.

Group			Active users, n (%)		Number of sessions over 6-week app trial				
					Mean (SD)		Median (IQR)		Top 25th percentile
All users			24 (100)		27.1 (47.5)		10 (7-18)		18
**Gender**									
	Women	15 (62.5)		37 (57.7)		10 (6-47)		47	
	Men	8 (33.3)		10.8 (4.5)		11 (7-16)		16	
**Recovery goal**									
	Abstinence recovery goals	14 (58.3)		13.5 (10.7)		10 (6-16)		16	
	Harm reduction recovery goals	10 (41.7)		46.2 (68.0)		14 (7-54)		54	
**Perceived app helpfulness**									
	Somewhat to very helpful	13 (54.2)		32.2 (57.4)		10 (6-18)		18	
	Somewhat to very unhelpful	9 (37.5)		24.2 (33.3)		15 (7-18)		18	
**Most problematic substance**									
	Alcohol is most problematic substance	9 (37.5)		34 (67.4)		9 (6-14)		14	
	Cannabis is most problematic substance	5 (20.8)		11 (3.7)		10 (9-11)		11	
	Other most problematic substance	10 (41.7)		29 (33.7)		16 (6-47)		47	
**Age group (years)**									
	18-29	5 (20.8)		9.8 (2.9)		10 (9-11)		11	
	30-39	14 (58.3)		38.1 (59.6)		10 (7-47)		47	
	40-59	4 (16.7)		12.5 (7.7)		15 (6-24)		24	
**Race and ethnicity**									
	White	14 (58.3)		36.4 (59.3)		15 (6-24)		24	
	BIPOC^a^	7 (29.2)		16.1 (16.0)		9 (6-18)		18	

^a^Black, Indigenous, and People of Color.

While much variation can be seen in the mean and 25th percentile values, the median number of sessions is relatively stable for most groups. This suggests that a small portion of users had a particularly large number of sessions that inflated the mean and percentile values.

**Figure 5:**
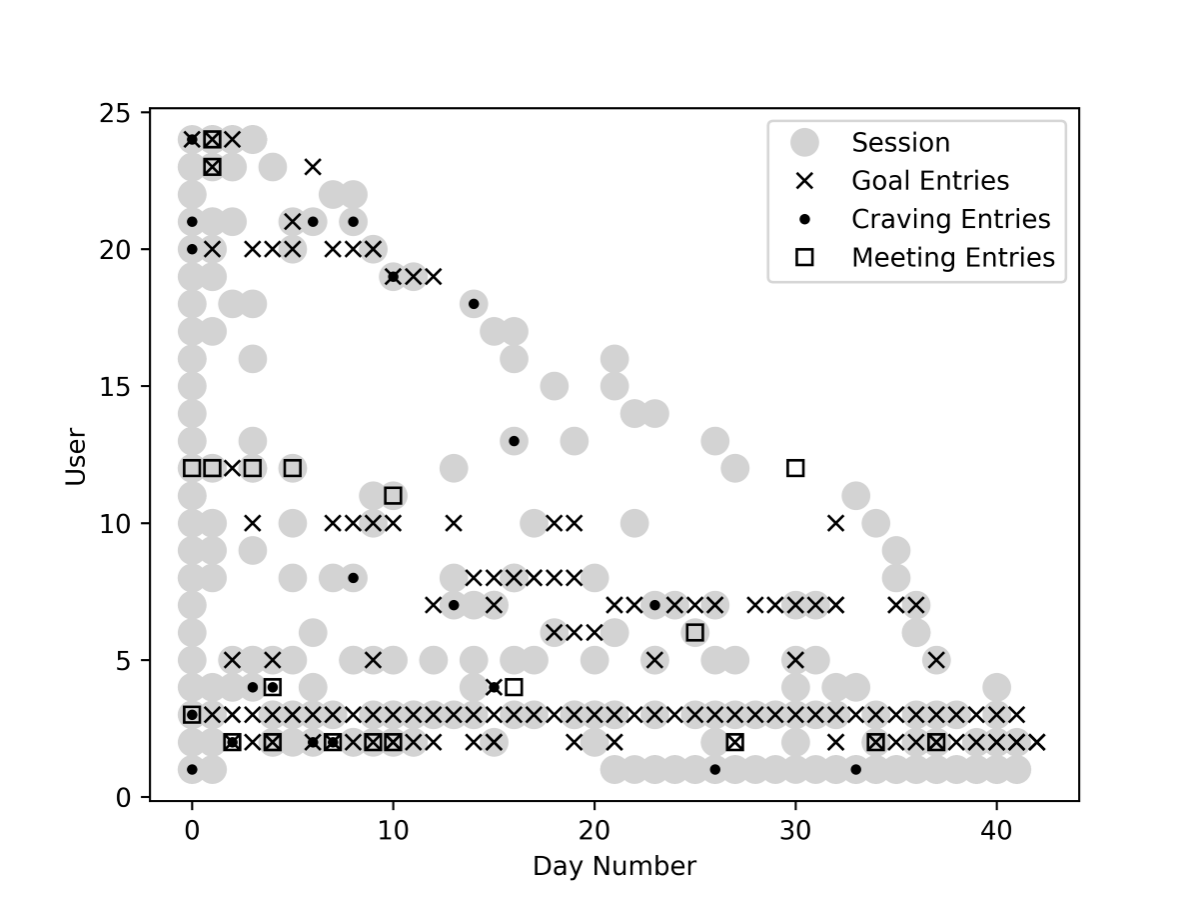
Each day in the trial when active users opened the app (sessions) and made new entries into each of the new co-designed features.

Figure 5 shows the temporal pattern of engagement with the app in general (sessions), as well as engagement with each of the 3 new features over the 42-day app trial. The x-axis represents the day number relative to each person’s first day in the trial, and the y-axis represents user indices. Note that the user indices in Figure 5 are randomly assigned to the data in Figure 5 only and do not correspond to the participant IDs used when reporting interview data.

To better observe trends, users are ordered in Figure 5 according to the last day they used the app. When creating a goal, craving, or meeting entry, the user had the option to manually adjust the date that the goal was met, cravings were experienced, or the meeting occurred. Therefore, the goal, craving, and meeting data points shown in Figure 5 are based on the dates manually entered by the user, rather than the dates that the user opened the app to enter the data. Furthermore, sessions (gray circles) reflect the exact dates that each user opened the app. During each session, users may have engaged with the myriad of preexisting Zamplo features, such as activity tracking, medication tracking, creating and viewing charts of personal data, and so on.

Figure 5 depicts the relative popularity of the Zamplo features co-designed during this study compared to users’ engagement with the rest of the Zamplo app. For example, users 15, 16, and 17 logged sessions of app usage but did not use the goals, cravings, or meeting features. In contrast, user 3 made nearly daily use of the app and the goal feature specifically. When viewing the full figure, it is clear that most users chose to interact with the newly designed goals, cravings, and meetings features. However, their use of these features varied widely, with some patterns of almost daily use and other patterns of intermittent and infrequent use.

The new goals feature was the most popular. More granular analysis of usage data revealed that, of the 24 active users, 17 created a single goal to track, 3 created multiple goals, and 4 did not create a goal. When users entered data into the app via the new co-designed features, 58.3% (14/24) made at least 1 goal entry, 50% (12/24) made at least 1 craving entry, and 33.3% (8/24) made at least 1 meeting entry.

A common metric for understanding app engagement is the 30-day retention rate, defined as the percentage of users still using the app 30 days after installing it. If all authentic users are included in this metric, 20.8% (11/53) of authentic users were retained after 30 days. This metric can also be calculated using only the active participants who made some effort to use the app, since the others may never have intended to use the app beyond minimal research compliance. In this case, 45.8% (11/24) of active participants were retained after 30 days.

### Phase 3: User Experience Interview Findings

#### Overview

Qualitative interviews contextualized the above quantitative findings by revealing details of how participants experienced Zamplo. A total of 12 individuals from the app trial attended a one-on-one interview with a researcher. The mean duration of interviews was 47.6 minutes, ranging from 38 to 59 minutes (9.5 total hours). Interview participants had a mean age of 32.9 (SD 7.38) years. Participant demographics are shown in Table 3.

**Table 3:** Characteristics of interview participants.

Measure				Value, n (%)
**Demographics**				
	**Gender**			
		Women	7 (58.3)	
		Men	5 (41.7)	
	**Age group (years)**			
		18-29	4 (33.3)	
		30-39	6 (50)	
		40-59	2 (16.7)	
	**Ethnicity**			
		White	7 (58.3)	
		BIPOC^a^	5 (41.7)	
**Substance use disorder history**				
	**Most problematic substance**			
		Alcohol	4 (33.3)	
		Prescription medications	4 (33.3)	
		Cannabis	2 (16.7)	
		Methamphetamine	1 (8.3)	
		Heroin, fentanyl, or other opioid	1 (8.3)	
	**Recovery approach**			
		Aim to reduce substance use (harm reduction)	7 (58.3)	
		Aim to stop substance use (abstinence)	5 (41.7)	
	**Time since taking first steps to address substance use disorder**			
		3-7 months	3 (25)	
		1-2 years	3 (25)	
		6-10 years	3 (25)	
		Unknown	3 (25)	
**Perceived app helpfulness**				
	Somewhat to very helpful		9 (75)	
	Somewhat to very unhelpful		3 (25)	

^a^BIPOC: Black, Indigenous, and People of Color.

During interviews, participants described their experiences with Zamplo and other digital recovery tools, and identified their needs for future mHealth apps based on their reflections from this study. Below are findings from across these domains, focusing first on results from preliminary codebook analysis and then on 3 emergent themes from the final thematic analysis.

Codebook analysis highlighted several app features that participants repeatedly spoke highly of, prompting ideas for future mHealth apps. Favorable features included goal-setting features, life-organization features (eg, appointments, medication reminders, and to-do lists), and self-tracking and visualization features. Interviewees described beneficial mental and behavioral outcomes of interacting with such features, which included the following, in descending order of salience in the interview data: increased motivation to self-improve, feelings of accountability, greater focus on healthy habits and distraction from unhealthy habits, increased self-awareness, a sense of self-empowerment, and increased self-compassion. Functionality that was not configured in Zamplo during this study but that participants enjoyed using in other apps included gamification features (eg, rewards for progress or accumulating “points”), information and resources (eg, personally-targeted mental health tips and other educational information), community and social features (eg, group forums, chats, webinars, and media-sharing), and inspirational content (eg, regular personal affirmations and motivational quotes).

Despite the positive outcomes described by interviewees, quantitative usage metrics painted a diverse picture of engagement. A closer thematic analysis of interview data adds nuance to participants’ experiences, helping contextualize our mixed usage metrics by highlighting what participants struggled with, what they would appreciate in future tools, and potential design challenges when creating an engaging mHealth app for recovery. The following sections describe three key themes: (1) a digital journal as a touchpoint to greater recovery resources, (2) a twofold experience of Zamplo’s open-ended configuration, and (3) expanding beyond substance use in recovery apps.

#### A Digital Journal as a Touchpoint to Greater Recovery Communities

Upon reflecting on their experiences during this app trial, participants tended to describe their needs along three categories of features, (1) personal features—journaling tools for individual self-monitoring, (2) relational features—active social spaces that enable interaction with surrounding recovery communities, and (3) informational features—frequently updating content for users to feel connected and informed about current conversations in recovery. The ideal app would bridge functionality between these 3 realms, not only providing a space for private reflection but also serving as a conduit to wider recovery ecosystems.

The first category (personal features) included goal-setting, tracking, and charting functionalities that allow participants to gain awareness and take ownership of their recovery. Such features were already configured in Zamplo during this study. The second category (relational features) included several tools that participants appreciated in other apps and would like to see integrated into a single recovery app, alongside the aforementioned personal features. These features included social networking and community spaces that allow users to share with and learn from others in recovery who are experiencing common struggles. For example, P071 requested organized peer support groups to be built into an app like Zamplo:

I think this is actually very important. I would actually want to have this connection with peer support groups and online community. I think that would actually be very helpful to have this online community and support groups actually around in the app.

P071

P032 similarly explained that their ideal recovery app would include the following:

…the possibility of chatting with other people experiencing the same symptoms or the same issues. Because, sometimes, it enhances the motivation, and you learn from other people's experiences.

P032

For relational features like these to function as participants have described, the platform would require a significant number of users who face similar challenges to join the platform so that they can begin to connect with one another. The final category (informational features) encompassed resources such as frequently-updated news postings about substance use and recovery, “tips and tricks” articles to support recovery and mental health, educational resources about substance use, inspirational stories or quotes, and personalized reading material. This type of content would provide users a sense of connectivity to the wider world of recovery. For example, P103 explained that it would be important for the app to frequently refresh with new recovery-focused content to serve as a positive pastime and increase app engagement:

I think I would have less anxiety, and I'd be happy to go into the app all the time, and just seeing what's new. Seeing if they added anything, or if there's any new articles or stuff to help you. Just maybe something new every day. And it's just, ‘oh, look at this,’ or, yeah. It just makes you happy.

P103

Zamplo, as it was configured during this study, did not provide this sense of connectivity with others in recovery nor did it offer personally-targeted, timely content to consume. It instead focused on providing a private digital space for participants to manage their personal recovery journeys. The minimal connection with the wider recovery world may have contributed to the mixed levels of engagement seen in quantitative usage metrics.

#### A Twofold Experience of Zamplo’s Open-Ended Configuration

Zamplo was configured for this study with an open-ended architecture that allowed for a self-guided user experience. As such, it did not enforce that users track any specific metrics, nor did it enforce any specific types of goals to be set, or nudge user interaction according to any preset schedule. Instead, it provided participants with the means to track whichever items, set whichever goals, and schedule whichever reminders they desired. Participants were informed upon signup that they were not required to interact with Zamplo in any particular way for this study and could use the app as much or as little as they wished. This not only allowed for more naturalistic observations of usage behavior, but also surfaced a tension in participants’ values regarding flexibility and control.

Zamplo’s flexibility gave many participants a sense of control over their own experience. For example, P054 explained, “I liked that it was very much ‘I took control of it,’ and I set my own goals, and there were lots of options and reminders, if I wanted to.” Similarly, P096 contrasted the flexibility of Zamplo with the rigidity of other recovery apps. Other apps often limited them to only tracking one recovery-specific goal, such as abstinence, triggering unhealthy responses when the goal is met.

The one [app] that was tracking my sober time, I would get too excited when it got to the month, and I'm like, “okay, let's go party.” ...In this one [Zamplo], I like how I could add things and take away things. Because the other one, for me, it was just one thing, and once I reached that [goal], I felt like I had to start fresh. And I didn't see my progress for the other things, so it's like, oh, well, let's go celebrate this one. And that celebration was not always a great celebration.

P096

Only allowing the user to perceive their progress according to one dimension of their recovery was detrimental to P096’s recovery. In contrast, Zamplo gave P096 the freedom to adapt their interface so it included a wide range of personalized goals to track.

P032 similarly described Zamplo’s open-ended design favorably, explaining that they could engage with the app “at [their own] pace.” P032 contrasted Zamplo with other apps that used a more strict, prescriptive approach, making them feel “pressured” if they did not adhere to the engagement behavior enforced by the app, such as preset daily tasks to complete. Participants who appreciated the freedom to build their own experiences in Zamplo tended to describe benefits such as increased accountability, enhanced motivation, a sense of empowerment, and heightened self-awareness.

This open-ended quality of Zamplo, however, also spurred negative user experiences. Some participants described feeling overwhelmed or confused by the interface, as it did not provide the direction they would have liked. P019 described the user experience as a “free-for-all”:

I feel like usually apps will kind of lead you through things in a certain way, and I feel like [Zamplo] was just a free-for-all. And so then it kind of just made me be like, “okay, too much going on.” And I really didn't use, I don't feel like, a lot of it.

P019

While Zamplo’s user-guided configuration gave some participants a sense of agency over their own recovery, this excerpt emphasizes that others felt burdened by the same functionality. Participants who did not appreciate Zamplo’s open-ended quality often preferred a preprogrammed, prescriptive user experience.

It could have been easier to be like, okay, maybe “What's your goal with the app?” or “What's your goal in the next month?” Or whatever. You [the user] could say “reduce using a substance,” or something. And then you could choose from a list it could prepare. ...And then it could show you, “okay, go reduce your drinks by this many,” or whatever. And then it would establish some tracking starter points based on that.

P034

Here, P034 has ideated a user workflow that offers suggestions for recovery strategies based on personalized questions, rather than fulfilling only a passive journal-like role, as Zamplo did.

P103 additionally recalled negative experiences attempting to understand the purpose of the app: “I didn't know, like, what the meaning of the app was. Without a tutorial or, like, what am I supposed to do?” Participants did receive a step-by-step tutorial when creating an account, which included a walkthrough of each feature available to them. Still, some participants were unsure how to begin their app experience without prescribed items to track or a preset workflow to adhere to. This suggests that either the tutorial did not meet participants’ needs or that they may not have engaged with it fully, possibly due to differing expectations of what a “recovery app” should provide.

A closer look at P019’s interview offers an example of this mismatch in expectations. They expected a more prescriptive app design and described their experiences with a local treatment center as context for how they would expect a recovery app to function.

I just think that the app should guide the person, more than the person going in and really guiding it themselves. ...When I was in [anon. treatment program], they do try to keep things very basic. It's like, they structure everything for you so that you can just focus on your recovery.

P019

Here, P019 explained that they would prefer an app that provides a close equivalent to a formal, in-person treatment program, structuring the user experience with a top-down plan. This contrasts others’ preferences for an app that allows them to set their own usage path with a multitude of options to complement in-person recovery rather than emulate it.

In summary, a tension arose from the open-ended configuration of Zamplo. The flexibility and nonprescriptive nature of Zamplo allowed some participants to feel in control of their own recovery. This led to fruitful interactions with the app’s tracking and goal features, which gave rise to feelings of accountability, motivation, empowerment, and self-awareness. Meanwhile, others felt burdened by the onus of creating their own recovery regimen in the app, causing overwhelm and confusion, which may have played a role in their disengagement.

#### Expanding Beyond Substance Use in Recovery Apps

Finally, while the previous theme pertained to the back-end configuration of Zamplo, this theme describes the front-end subject matter available within the app. Most participants appreciated the potential to use Zamplo for purposes outside of recovery. As P096 described, Zamplo encompassed all aspects of a person’s recovery journey.

I'm not just addiction. I'm not just mental health. I'm not just physical health. ...I am all of them, and they all impact me. Being hungry can impact my mental health, which could impact my triggers to wanting to use. It's all into one. ...This app is everything. It's not just recovery, it's not just mental health. This is a bit of everything.

P096

Furthermore, P054 used the goals feature to track goals not directly related to recovery, such as reading goals. They also used the goals feature to manage daily to-do items:

I just found it was just really easy to use and keep track of my goals, my expectations. I ended up using it for a lot of things, basically as my to-do list, and I really liked it.

P054

Notably, they still considered their reading goals and to-do items under the purview of “substance use” goals because they view their recovery as a holistic trajectory toward healthier daily habits. Similarly, P071 described using the fitness tracking features within Zamplo as their primary use case throughout the study, which they still considered part of their substance-use recovery regimen. The below excerpt exemplifies how this holistic app design led to a variety of tracking possibilities that all ultimately supported participants’ recovery:

I think playing the guitar, and gratitude journaling, fitness, using medication, I think these are the things I set aside for myself in the application [Zamplo] to carry out on a daily basis. And these are the things that actually help me to avoid the usage of substances.

P127

However, there was a trade-off to incorporating such multifunctional, holistic tracking functionality in Zamplo. This led to an overabundance of features on the interface, each with many potential use cases, again causing overwhelm for some participants.

Because I can get overwhelmed easily, and I don't like having too many options because I'm extremely indecisive, this was just way too busy for me. ...Honestly, I tried to not come into the app as frequently.

P019

Notably, though, those who disengaged due to feelings of overwhelm still described a preference for holistic app designs with a wide range of functionalities, rather than apps that narrowly target substance use behaviors. This preference came through in their descriptions of other apps that they have previously enjoyed and their vision of an “ideal” recovery app. Many explained that tools to assist with basic life maintenance, such as establishing daily routines or celebrating “small wins,” like doing household chores, are all crucial for their recovery. However, it is essential that the digital platform provides enough clear guidance to help them navigate through the potentially overwhelming quantity of available features.

## Discussion

### Overview

This study resulted in the design and deployment of 3 new mHealth app features: a goal-setting feature, a craving tracker, and a meetings log. Usage analyses and interviews foregrounded quantitative and qualitative findings regarding how participants engaged with and perceived these new features and the app in general. Overall, 45.3% (24/53) of participants opened Zamplo on at least 2 separate occasions other than the required first and last days of the trial period, deeming them “active” users. These 24 active users opened Zamplo 27.1 times on average over the 42-day trial, and 45.8% (11/24) of them were still using Zamplo after 30 days.

Interviews highlighted that participants desired tools that not only act as private digital recovery journals, but also as gateways to connect with wider recovery communities and as sources of engaging and educational recovery content. A design tension also arose between prescriptive user workflows, where the app guides the user through a structured recovery plan, and open-ended platforms, where users set their own recovery regimens. Lastly, interviewees largely appreciated when apps not only focused on recovery but also included features to support them in other dimensions of their lives.

Scheibein et al [40] proposed a roadmap to achieve optimal use of digital tools in the substance use field by 2030. They identified 5 key values based on a large backcasting exercise: digital rights, evidence-based tools, user-friendliness, access or availability, and person-centeredness. They additionally warned that current digital recovery tools are heavily informed by the interests of multinational, for-profit companies, with minimal involvement from key stakeholders such as people actively struggling with substance use disorders [40]. This study has described 3 phases of a co-design and deployment process, answering this call to include end users’ voices in technology design. Analysis led to several methodological insights and design implications that help to integrate the user-friendliness and person-centeredness values into recovery app development.

### Reflections on Methodology

This research exemplified an end-to-end app development and testing cycle, offering a real-world case study of iterative co-design and deployment with those in recovery. As researchers strive to optimize recovery app engagement, we urge that they incorporate co-design into their methods. Valuable insights were gained during each of the 3 co-design research phases in this study. Phase 1 provided actionable takeaways for app development based on what those with recovery experience described wanting in their recovery platforms, leading to a concrete starting point for new features. In Phase 2, we gathered real-world engagement based on the features from Phase 1, revealing complex usage patterns as some users engaged and others disengaged from the app. Usage analysis then gave way to Phase 3 interviews, where nuanced design tensions came forward during one-on-one conversations with participants who could now draw upon their hands-on experience with the app.

Each phase was critical for the next phase, and they all contributed to understanding participants’ perspectives on how to increase app engagement and support this population. These phases represent the beginning of a deeply iterative co-design process in which practitioners investigate end users' needs, translate their ideas into concrete tools deployed for usage, and reflect with users on the real embodiments of their original ideas. This last stage can then prompt further dialogue about future iterative designs to continue aligning more closely with users’ needs.

Given the relatively low extant evidence of uptake and engagement for recovery apps, amplifying the perspectives of people with lived experience may be key to understanding why they may or may not engage with these apps. Although not all participants engaged deeply with the app during this study, still 20.8% (11/53) of all users were retained after 30 days, and 45.8% (11/24) of active users—those who went beyond minimal compliance for this study—remained engaged after 30 days. By industry standards, the typical 30-day retention rate in 2024 was 7% for all mobile apps [41] and approximately 4% for digital health apps [42]. The substantially higher retention rate in this study could be explained by participants being incentivized to take part. However, we mitigated this bias by repeatedly stating that participants are not required to interact with the app during the study to receive their incentive and are instead encouraged to use it however frequently feels helpful for them. These retention figures suggest that Zamplo features can be taken as a promising starting point for future recovery app designs. Additionally, the co-design approach may have contributed to engagement, though further controlled studies are needed to confirm this effect. This finding parallels prior literature concluding that incorporating end users’ perspectives into app design can increase uptake and engagement [12]. Importantly, though, a portion of participants still disengaged from the app, necessitating further discussion of design tensions, challenges, and opportunities for future platforms.

### Implications for Design

This research surfaced several design trade-offs that are crucial to consider when designing engaging mHealth apps for recovery. Interviews revealed a clear split in positive and negative experiences with Zamplo: positive experiences arose from feelings of agency and autonomy over one’s own recovery, while negative experiences manifested in feelings of overwhelm and confusion. Both types of experiences resulted from the same open-ended, holistic, and flexible nature of Zamplo. As per prior research, mHealth interventions are not likely to be “one size fits all” [7]; users’ needs often vary, and rigid designs are not likely to appeal to all members of the heterogeneous group of individuals in recovery. This study sheds more light on this diversity of experience and urges future designers to strike a balance between system-controlled versus user-controlled design approaches.

This tension echoes a longstanding debate in user experience research regarding the extent to which the system should impose predetermined, stepwise flows onto users, versus letting users freely set their own app workflows. While this tension is well explored in general user experience research and persuasive design domains [43], it has not been explicitly foregrounded in substance use recovery technology research, as this study has done. Indeed, this debate is particularly interesting within the recovery domain, where self-tracking technologies are often packaged as prescribed programs with fixed tracking metrics, similar to how counselors provide trusted top-down structures and routines for patients to follow. Preliminary studies in the field of human–computer interaction, however, show that app users in recovery may in fact appreciate more holistic and flexible self-tracking [25,29].

This study extends this line of inquiry by empirically demonstrating this trade-off as it relates to the recovery domain. One possible way to reconcile this tension is to design recovery apps that include both a ground-level “starter kit” with recommended features and evidence-based usage routines, while also offering additional, optional, and customizable features built on top of starter features. Users like P019, who feel overwhelmed by too much freedom, could benefit from a set of default features that reduce cognitive load and help them jumpstart their digital recovery journey. These default features could operate as a digital analogue to structured treatment programs with planned recovery pathways. For example, they could include preset goals with rigid tracking schedules, preset daily self-monitoring routines including prompts for regular check-ins and written reflections, and specific personal metrics to track on a set schedule, such as substance use quantity per day or recurring triggers. This design would need to explicitly guide the user through the interface, instructing them on how to use each feature to support their recovery, similar to a counselor’s prescriptive guidance. This may be most useful for those who are new to using digital recovery apps.

Then, users like P032, who are confident to carve their own recovery pathways in a digital platform, could personalize the app to step outside the prescribed usage flows and ensure they feel in control of their recovery. This optional extended functionality could offer, for example, highly flexible goal setting with customizable goal timelines, monitoring of any chosen personal metrics, and user-determined reminders and check-in schedules to enhance self-reflection at a pace the user is comfortable with. It would be crucial for these additional features to allow tracking outside of substance-use itself to align with the present findings and the broader literature suggesting a holistic approach to recovery [29,44-46].

The distinct split between positive and negative experiences with Zamplo additionally raises questions about the characteristics of the individuals in each category. Milward et al [18] developed typologies of user engagement with harm reduction apps. They identified “trackers” who liked to track several metrics for the sole purpose of self-awareness; “cut-downers,” who focused on finding a solution to their substance use and made more use of goal-setting features than self-tracking features; and “noncommitters,” whose initial enthusiasm about the app quickly waned and led to disengagement.

This study found similar patterns, as illustrated in Figure 5. Several participants used Zamplo’s existing tracking features but did not engage heavily with the goal-setting feature (trackers); a portion of participants displayed particularly high usage of the app in general, with special attention paid to the goal-tracking feature (cut-downers); and a portion of participants lost interest in the app after signing up for the study and never became an active user or they only engaged during the beginning of their trial period (noncommitters).

This study extends this characterization by placing more onus on designers rather than users, and suggesting that the noncommitters category does not reflect a homogenous group of people who are generally disinterested in trying to use recovery apps. We propose that this typology can be split into 2 subcategories based on the user’s compatibility with the app design. One subcategory is reserved for people who are, indeed, not committed to using apps for recovery. We call this subcategory “true noncommitters.” We refer to the second subcategory as “unintentional noncommitters.” This group includes those who are indeed committed to finding digital recovery solutions but may lose interest in particular platforms due to ineffective designs that do not align with their nuanced recovery needs, as unpacked during this research.

### Limitations and Future Work

Using existing Zamplo architecture provided us with the resources to conduct an extensive real-world app deployment study. It also provided insight into how recovery-focused features can integrate into an established mHealth platform and coexist with features not directly related to recovery. However, the preexisting Zamplo features were not the result of our co-design with end users, yet were still included in participants’ experiences. This limits the internal validity of our evaluation, specifically of the co-designed features, because user experiences with those features may have been confounded by interactions with the preexisting functionalities of the platform. Additionally, parts of what participants in co-design sessions requested were not able to be fully embodied by the new co-designed features due to resource constraints and because the features had to integrate into existing Zamplo architecture. Fraudulent participation also posed issues for this study, decreasing sample sizes in the later phases of the work. These sample sizes reduced the transferability of these findings. However, this study still highlighted several noteworthy emergent usage patterns and design tensions, despite small sample sizes. Future larger-scale research endeavors with strategies to eliminate fraudulent participation are necessary to confirm the salience of our findings.

A key finding of this study was the diversity of experience and attitudes toward using the app, as highlighted in the results of Phase 3. The small sample size of Phase 3 prevented analysis that would determine if demographics (eg, age and gender), or clinical factors (eg, recovery stage) can be linked to values and attitudes toward using a recovery app (eg, valuing autonomy versus a more prescribed user experience). Future larger-scale studies should explore such correlations in more detail. This study did reveal a subset of users inclined to feel overwhelmed by all of the available features in the Zamplo platform. Therefore, future studies using general-purpose health platforms for addictions recovery could also explore mitigating overwhelm via providing extra tutorial material.

Finally, it is important to note that this study’s prime focus was capturing realistic measures of engagement with an mHealth app for recovery. However, prior work has brought into question the engagement-efficacy perspective, which assumes that higher engagement with health technology is a precursor to long term efficacy [17]. Instead, Smith et al [17] identified “productive disengagement,” a phenomenon where disengaging with a health app can actually help the user in achieving their health goals. This study was unable to pinpoint every participant’s exact reasons for disengagement, and instead characterized any disengagement as a negative result. As future research works to optimize engagement with recovery apps, it is important to acknowledge the engagement–efficacy assumption so that higher engagement is not always conflated with higher efficacy. Future work may benefit from investigating further into specific users who disengage with recovery apps to understand the details of their disengagement before necessarily classifying it as a negative result.

### Conclusions

This study presents a detailed account of incorporating end users’ design ideas into new app features to investigate engagement. We collaborated with the mHealth platform, Zamplo, to deploy 3 new app features: a goal tracker, a craving tracker, and a meeting tracker. A 6-week app trial revealed mixed engagement with the app, with 30-day retention rates substantially higher than industry benchmarks. Qualitative analysis foregrounded several mHealth features that participants perceived as valuable for their recovery. Difficult design tensions also came forward, such as balancing system control with user autonomy while mitigating cognitive load for users, as well as integrating private journal-like features into apps that also connect users to wider recovery ecosystems. This study offers early insight into the value of participatory design methods with real-world app deployment for understanding digital recovery engagement, highlighting directions for future mHealth research and design.

## Appendix

Multimedia Appendix 1 Qualitative codebook used by two independent coders to analyze user experience interviews.

## Abbreviations

CONSORT: Consolidated Standards of Reporting Trials
mHealth: mobile health
RtD: research through design
