# Supplementary material for: Designing mHealth Apps for Substance Use Recovery Through Real-World Co-Design and Deployment: Mixed Methods Study
Source: JMIR Mhealth Uhealth. 2026 Feb 11;14:e83984. doi: 10.2196/83984 (PMC12936659; doi:10.2196/83984)
Supplement: Multimedia Appendix 1 [file mhealth_v14i1e83984_app1.docx]

The following codes are organized into a hierarchy, and all are included in the NVivo project (.nvp file). The corresponding code descriptions are also included in the NVivo project.

To see the description of each code in NVivo: select the code, right click, and go to Code Properties. Alternatively, select the code, and use the keyboard shortcut Ctrl+Shift+P. The character limit for the NVivo code descriptions were not large enough for some descriptions. For those codes, I included a note in the description to refer back to this document.

**EXISTING TECHNOLOGIES**

This is only a header for codes, not a code itself. All discussion of existing technology should be coded under either the *Recovery-Specific Technologies* code or the *Not Recovery-Specific Technologies*.

| **Code** | **Description** |
| --- | --- |
| **Recovery-Specific Technologies** | All discussion of existing recovery-specific technology should be coded here.   - Any mention of an existing piece of technology that is intended to support recovery. For example: *I Am Sober* app, *Sober Buddy* app, *Less* app - Descriptions of experiences with the existing technology, such as what the participant liked or did not like about it. |
| **Not Recovery-Specific Technologies** | All discussion of existing general technology should be coded here.   - Any mention of an existing piece of technology that is not necessarily intended to support recovery. For example: fitness apps, general health apps, ChatGPT. - Descriptions of experiences with the existing technology, such as what the participant liked or did not like about it. |
| **Memorable Quotes** | If you come across any interview excerpts you find particularly impactful that are related to experiences with existing technology, save them by coding them here. |

**PARTICIPANT’S TECHNOLOGY NEEDS & IDEAS**

This is only a header for codes, not a code itself. All mentions of technological features and designs should be coded under one or more of the subcodes below

| **Code** | **Description** |
| --- | --- |
| **Goal Setting** | Include all mentions of goal setting, including long and short term goals, recovery and non-recovery goals, and any other discussion of goal setting.  Include all mentions of goal setting regardless of if they pertain to an existing technology, Zamplo, or hypothetical ideas for future technologies, and regardless of associations with positive or negative feelings/behaviours.   - If the excerpt pertains to an existing technology, double-code under the appropriate *Existing Technologies* subcode. If it pertains to Zamplo, double-code under the appropriate *Zamplo-Specific Feedback* subcode. - If the excerpt is associated with positive feelings/behaviours, double-code under the appropriate *Potential Positive Feelings* subcode. If it is associated with negative feelings, double-code under the appropriate *Potential Negative Feelings* subcode.   Include all mentions of goal setting, even if they seem to not directly relate to technology. Although this is rare since all interviews are in the context of technology, general personal experiences are still important to capture when investigating how technologies do or do not meet participants’ needs. |
| **Information & Resources** | Include all mentions of accessing information or resources such as educational content, news, blogs, or other written, audio, or visual content.  Similar to above, include all discussion of access to information & resources, double coding when applicable. |
| **Self-Tracking & Charting Personal Data** | Include all mentions of tracking or charting one’s personal data, such as keeping track of behaviours or moods, graphing or charting personal variables over time, or noticing patterns or overlaps between various aspects of one’s life.  Similar to above, include all discussion of self-tracking/charting personal data, double coding when applicable. |
| **Day-to-Day Life Organization**  **(i.e. Appointments, Medications,**  **To Dos)** | Include all mentions of staying on top of tasks, chores, or other day-to-day needs, such as appointments, medications, or to do items.  Similar to above, include all discussion of day-to-day life organization, double coding when applicable. |
| **Community & Social Features** | Include all mentions of interacting with other people via technology, such as direct messaging, sharing content, group interactions, or other social interactions.  Similar to above, include all discussion of community and social features, double coding when applicable. |
| **Inspirational Content** | Include all mentions of seeing inspirational or motivational content, such a positive affirmations, or any other written, audio, or visual content intended to inspire or motivate.  Similar to above, include all discussion of community and social features, double coding when applicable. |
| **Notification Preferences** | Include all mentions of technological notifications or reminders, including preferences for frequency, purpose, or any other aspects of notifications or reminders.  Similar to above, include all discussion of community and social features, double coding when applicable. |
| **Gamification** | Include all mentions of gamification, such as accumulating points, climbing “levels,” or any other game-like experiences.  Similar to above, include all discussion of community and social features, double coding when applicable. |
| **Personalization, Adaptability, Flexibility** | Include all mentions of the personalization/customizability, adaptability, or flexibility of technology, such as having the technology adjust to the user’s needs or circumstances, customizing the aesthetics of an interface, or having specific features change based on what different individuals prefer.  Similar to above, include all discussion of personalization, adaptability, and flexibility, double coding when applicable. |
| **Other Technology Needs & Ideas** | In this code, include any other discussion of technological features that are not already captured in the above codes. This could include participants’ specific ideas for new features, or abstract ideas for general technological experiences. This may also include descriptions of what participants prefer to *not* see in their technology. |
| **Memorable Quotes** (add annotation to specify the related sub-code) | If you come across any interview excerpts you find particularly impactful that are related to participants’ technological needs or ideas, save them by coding them here. Use NVivo to create a new “Annotation”, to specify the particular subcode (goal setting, gamification, etc.) the quote pertains to. |
| **HOLISTIC APPROACH TO RECOVERY**  This is a sub-header for the below codes. | |
| **Technology Should Consider Recovery as Part of Overall Health** | Any preference **for** a more “holistic” approach to recovery should be coded here. This includes preferences for tools to not only support recovery, but also support other non-recovery aspects of one’s life, such as physical or mental health. |
| **Technology Should Address Recovery Separately from Overall Health** | Any preference **against** a “holistic” approach to recovery should be coded here. This includes preferences for recovery-specific tools to *only* support recovery, while other non-recovery aspects of one’s life are addressed separately, using other tools. |
| **Memorable Quotes** | If you come across any interview excerpts you find particularly impactful that capture preferences for or against a “holistic” approach to recovery, save them by coding them here. |
| **SELF-DIRECTED VS PRE-PROGRAMMED APP EXPERIENCE**  This is a sub-header for the below codes. | |
| **Preference for Self-Directed App Experience** | Any preference for technology to act as a “blank slate” for the user to set up their own experiences, should be coded here. For example, if the participant appreciated that Zamplo did not have any pre-programmed goals, routines, or other features, and they found it easy to set up for themselves, they would have a preference for a “self-directed” experience.  Double-code when applicable with other technology needs/ideas codes, positive or negative feelings/behaviours codes, or Zamplo feedback codes. |
| **Preference for Pre-Programmed App Experience** | Any preference for technology to provide more focused direction and pre-programmed features for the user, should be coded here. For example, if Zamplo’s open-ended design came across as confusing, unfinished, challenging, overwhelming, or otherwise negative for the participant, they would have a preference for a “pre-programmed” app experience.  Double-code when applicable with other technology needs/ideas codes, positive or negative feelings/behaviours codes, or Zamplo feedback codes. |
| **Memorable Quotes** | If you come across any interview excerpts you find particularly impactful that capture preferences for or against a “holistic” approach to recovery, save them by coding them here. |

**POTENTIAL POSITIVE FEELINGS & BEHAVIOURS ASSOCIATED WITH TECHNOLOGY USE**

This is only a header for codes, not a code itself. All mentions of positive feelings or behaviours associated with technology use should be coded under one or more of the subcodes below.

Include any mention of feelings or behaviours resulting from technology use, regardless of the participant refers to an actual past experience with an existing technology or with Zamplo, or if it is based on a hypothetical scenario.

We will disambiguate by double coding to indicate if the experience relates to Zamplo feedback or an existing technology. Excerpts may also be double coded if they pertain to specific technology needs or ideas. An example of multi-coding can be found in the Overview doc.

| **Code** | **Description** |
| --- | --- |
| **Accountability** | Any descriptions of a sense of being held accountable to oneself or others, as a result or potential result of using technology, should be coded here. |
| **Empowerment** | Any descriptions of a sense of empowerment (including feelings of responsibility, control over one’s circumstances, maturity, self-efficacy), as a result or potential result of using technology, should be coded here. |
| **Motivation & Staying Focused** | Any descriptions of feeling or behaving motivated, or staying focused on what one wishes to stay focused on (such as personal goals, tasks, or healthy behaviours), as a result or potential result of using technology, should be coded here. Mentions of technology use feeling “rewarding” would be coded here as well. |
| **Self-Awareness** | Any descriptions of a sense of self-awareness, for example by noticing personal trends or behaviour patterns, as a result or potential result of using technology, should be coded here. |
| **Self-Compassion** | Any descriptions of a sense of self-compassion, such as reminding oneself to “take it one day at a time”, or emphasizing one’s own strengths and accomplishments over their downfalls, as a result or potential result of using technology, should be coded here. |
| **Helpful Recovery-Specific Outcomes** | Any descriptions of feelings or behaviours specifically related to positive/helpful recovery outcomes, as a result or potential result of using technology, should be coded here.  Excerpts coded here may often be double-coded under other feelings & behaviours codes as well. This code is meant to capture excerpts when participants tie their feelings or behaviours directly back to their recovery.  For example: A participant describes that a technology keeps them focused on their goals, which ultimately distracts them from substance use. This would be coded here as well as under the *Staying Focused* subcode. |
| **Other Positive Feelings & Behaviours** | In this code, include any other descriptions of positive feelings or behaviours that are results or potential results of using technology, that are not already captured in the above codes. |
| **Memorable Quotes** (add annotation to specify the related sub-code) | If you come across any interview excerpts you find particularly impactful that are related to participants’ positive feelings or behaviours, save them by coding them here. Use NVivo to create a new “Annotation”, to specify the particular subcode (accountability, etc.) the quote pertains to. |

**POTENTIAL NEGATIVE FEELINGS & BEHAVIOURS ASSOCIATED WITH TECHNOLOGY USE**

This is only a header for codes, not a code itself. All mentions of negative feelings or behaviours associated with technology use should be coded under one or more of the subcodes below.

Similar to the previous section, include any mention of feelings or behaviours resulting from technology use, regardless of the participant refers to an actual past experience with an existing technology or with Zamplo, or if it is based on a hypothetical scenario. Double code when applicable.

| **Code** | **Description** |
| --- | --- |
| **Overwhelm** | Any descriptions of a sense of overwhelm, as a result or potential result of using technology, should be coded here. |
| **Stigma** | Any descriptions of feeling stigmatized, including feeling shameful or at risk of judgement, as a result or potential result of using technology, should be coded here. |
| **Discouragement & Demoralization** | Any descriptions of a sense of discouragement or feeling demoralized, such as feeling weighed down or disheartened, as a result or potential result of using technology, should be coded here. |
| **Feels like “Work” & Lack of Motivation** | Any descriptions of a sense of excess effort or work required to use technology, with minimal reward or motivation from using technology, should be coded here. |
| **Detrimental Recovery-Specific Outcomes** | Any descriptions of feelings or behaviours specifically related to detrimental/harmful recovery outcomes, as a result or potential result of using technology, should be coded here.  Excerpts coded here may often be double-coded under other feelings & behaviours codes as well. This code is meant to capture when participants tie their feelings or behaviours directly back to their recovery. |
| **Other Negative Feelings & Behaviours** | In this code, include any other descriptions of negative feelings or behaviours that are results or potential results of using technology, that are not already captured in the above codes. |
| **Memorable Quotes** (add annotation to specify the related sub-code) | If you come across any interview excerpts you find particularly impactful that are related to participants’ negative feelings or behaviours, save them by coding them here. Use NVivo to create a new “Annotation”, to specify the particular subcode (accountability, etc.) the quote pertains to. |

**ZAMPLO-SPECIFIC FEEDBACK**

This is only a header for codes, not a code itself. All descriptions of positive and negative feedback specifically for the Zamplo app should be coded under one of the subcodes below.

Since the below subcodes do not capture what the specific feedback is (only if it was positive or negative), excerpts coded here will often be double-coded elsewhere, such as under positive or negative feelings/behaviours, or technology needs/ideas. Examples of excerpts that may not be double-coded elsewhere are statements about Zamplo that are vague and general, such as “I liked it” (positive), or “it wasn’t for me” (negative).

| **Code** | **Description** |
| --- | --- |
| **Positive Feedback** | Any excerpts where a participant speaks highly of the Zamplo app should be coded here. This includes brief statements about how participants positively experienced Zamplo, as well as longer descriptions of their positive opinions of the app.  As long as the participant is speaking favorably and referring specifically to Zamplo in their statements, rather than a different existing technology or a hypothetical idea for a future technology, then the excerpt should be coded here. |
| **Negative Feedback** | Any excerpts where a participant does not speak highly of the Zamplo app should be coded here. This includes brief statements about how participants negatively experienced Zamplo, as well as longer descriptions of their negative opinions of the app.  As long as the participant is speaking unfavorably and referring specifically to Zamplo in their statements, rather than a different existing technology or a hypothetical idea for a future technology, then the excerpt should be coded here. |
| **Memorable Quotes** | If you come across any interview excerpts you find particularly impactful that capture positive or negative experiences with Zamplo, save them by coding them here. |

**“SEEMS IMPORTANT” BUCKET**

This code is here to catch any excerpts that seem to be important in the big picture of this research, but the current arrangement of the codebook does not allow them to be captured. Anything that is not currently coded, but may lead to interesting findings, should be coded here. This code may lead discussion of codebook refinements.
